# Acute and chronic complication profiles among patients with chronic kidney disease in Alberta, Canada: a retrospective observational study

**DOI:** 10.1186/s12882-024-03682-z

**Published:** 2024-07-29

**Authors:** David C. W. Lau, Eileen Shaw, Suzanne McMullen, Tara Cowling, Kelcie Witges, Efrat L. Amitay, Dominik Steubl, Louis P. Girard

**Affiliations:** 1 Division of Endocrinology and Metabolism, Department of Medicine, University of Calgary Cumming School of Medicine, Calgary, AB Canada; 2 Medlior Health Outcomes Research Ltd, Suite 300 – 160 Quarry Park Blvd. SE, Calgary, AB Canada; 3 Boehringer Ingelheim International GmbH, Ingelheim am Rhein, Germany; 4 Department of Nephrology, Hospital rechts der Isar, Technical University Munich, Munich, Germany; 5 Division of Nephrology, Department of Medicine, University of Calgary Cumming School of Medicine, Calgary, AB Canada

**Keywords:** CKD, Comorbidities, Complications, ASCVD, Type 2 diabetes

## Abstract

**Background:**

Chronic kidney disease (CKD) poses a substantial burden to individuals, caregivers, and healthcare systems. CKD is associated with higher risk for adverse events, including renal failure, cardiovascular disease, and death. This study aims to describe comorbidities and complications in patients with CKD.

**Methods:**

We conducted a retrospective observational study linking administrative health databases in Alberta, Canada. Adults with CKD were identified (April 1, 2010 and March 31, 2019) and indexed on the first diagnostic code or laboratory test date meeting the CKD algorithm criteria. Cardiovascular, renal, diabetic, and other comorbidities were described in the two years before index; complications were described for events after index date. Complications were stratified by CKD stage, atherosclerotic cardiovascular disease (ASCVD), and type 2 diabetes mellitus (T2DM) status at index.

**Results:**

The cohort included 588,170 patients. Common chronic comorbidities were hypertension (36.9%) and T2DM (24.1%), while 11.4% and 2.6% had ASCVD and chronic heart failure, respectively. Common acute complications were infection (58.2%) and cardiovascular hospitalization (24.4%), with rates (95% confidence interval [CI]) of 29.4 (29.3–29.5) and 8.37 (8.32–8.42) per 100 person-years, respectively. Common chronic complications were dyslipidemia (17.3%), anemia (14.7%), and hypertension (11.1%), with rates (95% CI) of 11.9 (11.7–12.1), 4.76 (4.69–4.83), and 13.0 (12.8–13.3) per 100 person-years, respectively. Patients with more advanced CKD, ASCVD, and T2DM at index exhibited higher complication rates.

**Conclusions:**

Over two-thirds of patients with CKD experienced complications, with higher rates observed in those with cardio-renal-metabolic comorbidities. Strategies to mitigate risk factors and complications can reduce patient burden.

**Supplementary Information:**

The online version contains supplementary material available at 10.1186/s12882-024-03682-z.

## Three Key Points

• CKD poses a substantial burden to individuals, caregivers, and healthcare systems with respect to adverse events and conditions that occur with or following CKD.

• This study provides an overview of comorbidities/complications observed in patients with CKD in a Canadian real-world clinical setting. This report is more detailed than currently available published literature, examining a comprehensive list of cardiovascular, diabetic, renal, fracture, and infectious outcomes.

• CKD affects a substantial proportion of the adult population in Alberta, with high rates of complications experienced by patients with CKD, particularly among those with cardio-renal-metabolic comorbidities.

**Clinical implications**: Results from this study provide estimates to inform the healthcare resources needed to provide optimal care for patients living with CKD, including early detection and management of complications.

**Supplementary Information:**

The online version contains supplementary material available at 10.1186/s12882-024-03682-z.

## Background

Chronic kidney disease (CKD) poses a significant burden to individuals, caregivers, and healthcare systems. The estimated prevalence of CKD in Canadian primary care settings was recently estimated as 71.9 per 1,000 persons [1]. CKD is characterized by a reduction in glomerular filtration rate (GFR) and/or increased albumin excretion in the urine and is commonly associated with a loss of renal function over time [2]. CKD has been ranked the 8th leading cause of death in Canada as of 2019 [3]. 

Stages of CKD severity are classified based on estimated GFR (eGFR), with higher stages of CKD (stages 4–5) associated with greater morbidity, mortality, and healthcare costs compared to earlier stages (stages 1–3) [4–6]. A recent study conducted in Alberta, Canada (Manns et al., 2019) estimated the average one-year direct medical costs of approximately $91,100 Canadian dollars (CAD) for a patient with CKD who is receiving dialysis compared to $14,600 CAD on a patient with CKD who is not receiving dialysis [7]. The substantial morbidity, mortality, and healthcare costs suggest timely care for CKD patients aimed at slowing progression of the disease is crucial for decreasing the burden of disease.

CKD risk factors are similar to those associated with cardiovascular disease including, older age, smoking, hypertension, obesity, and type 2 diabetes mellitus (T2DM) [8]. Approximately 30–40% of patients with T2DM also have CKD [9–11]. Both CKD and T2DM are associated with an higher risk of atherosclerotic cardiovascular disease (ASCVD); it is estimated that 36% of patients with ASCVD also have CKD [12]. Moreover, CKD is associated with a 10–30 times higher risk of cardiovascular morbidity and mortality compared to the general population [2] Mortality risk is also inversely associated with GFR values, mortality being highest in stage 5 CKD patients [13]. It is estimated that hypertension occurs in approximately 23% of individuals without CKD however, as both a cause and consequence of CKD, approximately 36% of stage 1, 48% of stage 2, 60% of stage 3, and 85% of stage 4/5 patients have hypertension [14]. 

In addition to chronic complications, CKD can also lead to various acute complications, particularly as the disease progresses to later stages. These complications often arise due to the kidney’s diminished ability to maintain homeostasis and manage waste products, electrolytes, and fluid balance. Acute complications include hyperkalemia (elevated potassium levels in blood), [15] acute kidney injury (on top of CKD), [16] infections (due to immune system dysfunction, particularly in patients undergoing dialysis), [17–19] and heart failure (as a result of fluid retention or hypertension) [20]. 

Early detection and treatment of CKD slow progression, reduce complications, and improve an individual’s overall quality of life [21]. Despite availability of guidelines for CKD [22–24] screening and treatment, significant variability among healthcare providers in adopting and implementing guidelines remains a burdensome challenge [21]. A recent report highlights the need for more targeted guidelines for treatment of CKD in high-risk patients; specifically, CKD guidelines that target persons aged 60 years or older, with T2DM, hypertension or cardiovascular disease, or a family history of kidney failure [21]. Enhanced characterization of the distribution of comorbidities and complications (both acute and chronic) associated with CKD within these target population will support implementation of more CKD guidelines that assess and serve the individual’s most at risk for CKD progression and CKD-related complications in Canada.

The purpose of this research study was to describe the comorbidities and complications, both acute and chronic, among patients with CKD (by stage 1 through 5) and among subgroups of patients with and without a history of ASCVD and T2DM in the 2 years prior to entering the study cohort.

## Methods

### Study design & data sources

A retrospective observational cohort study was conducted using province-wide administrative health data in Alberta, Canada, which covers publicly-funded programs and services for all Albertan residents, regardless of private insurance status. The administrative health datasets used in this study are outlined in Supplementary Table 1. Research ethics board approval was obtained from the Health Research Ethics Board of Alberta – Community Health Committee. Authors used the STrengthening the Reporting of Observational studies in Epidemiology (STROBE) statement in writing this report [25]. 

### Study population

The study population included adult Albertan residents (≥ 18 years of age) with CKD identified between April 1, 2010, and March 31, 2019 based on a previously reported algorithm with both positive predictive value and sensitivity + ≥ 70% [26], requiring one of the following conditions: (1) one inpatient hospitalization (glomerular diseases, renal tubulo-interstitial diseases, renal failure, and urolithiasis) identified using International Statistical Classification of Diseases and Related Health Problems, 10th Revision (ICD-10-CA) codes, (2) three practitioner claims within one year using International Classification of Diseases, Ninth Revision, Clinical Modification (ICD-9-CM) codes or (3) a mean eGFR < 90 mL/min*1.73m^2^ or mean albuminuria ≥ 30 mg/mmol over 12 months (details provided in outlined in Supplementary Table 2). eGFR and albuminuria thresholds were based on the mean of two consecutive tests at least 90 days apart within a 12-month period. The CKD index date was the date of the first CKD record or abnormal laboratory value meeting the algorithm criteria. CKD stage was determined by eGFR/albuminuria cut-off values published in The Kidney Disease: Improving Global Outcomes (KDIGO) 2012 Clinical Practice Guideline for the Evaluation and Management of Chronic Kidney Disease (CKD) (Supplementary Table 3 and Supplementary Table 4) [27]. Patients were classified as stage 1, stage 2, stage 3a, stage 3b, stage 4, and stage 5 CKD based on laboratory values available at index. If a patient was unable to be staged based on the guidelines, they were classified as “no stage”.

### Study cohort

To identify comorbidities identified on or prior to index date and complications occurring post-index, the overall CKD study cohort was limited to newly identified patients (burden of illness cohort), utilizing a two-year lookback window, and at least one year of follow-up data following their index date (follow-up data to March 31, 2020 were available; laboratory data available to March 31, 2019). Patients that moved out of the province within one year of their index date were excluded from the study cohort. The index date of this cohort is anticipated to be a close approximation for diagnosis.

### Study variables

Patient characteristics captured at index date included age, sex, Alberta Health Services geographic zone, and mean follow-up time. Laboratory values (most recent) in the 1 year prior to and including index date were reported for hemoglobin A1C and albumin: creatinine ratio (ACR) values. The Charlson Comorbidity Index (CCI), a method for categorizing comorbidities of patients based on diagnostic codes within administrative data, was derived based on diagnostic codes from inpatient hospitalizations and practitioner claims in the one year prior to the index date [28]. The CCI is based on the presence of at least one diagnostic code, with different weights applied to different conditions to derived the score. The presence of comorbid conditions of interest (including ASCVD [29] and T2DM status at baseline) were defined up to two years before the CKD index date, based on diagnostic codes from published algorithms (Supplementary Table 5, Supplementary Table 6, and Supplementary Table 7). Complications of interest were identified following index date and included cardiovascular, diabetic, renal, and other complications (e.g., infections, fractures, anemia, and all-cause mortality; Supplementary Table 7). Complications occurring after the CKD index date could also be present as comorbidities (in the two years prior to CKD index date), as the analyses were intended to indicate when the conditions of interest were being captured. Moreover, chronic complications could only be identified once for each patient whereas, acute complications could be identified multiple times for each patient.

### Statistical methods

Patient characteristics of the newly identified cohort were analyzed descriptively including frequencies, proportions, means, and standard deviations (SD). Comorbidities were described as frequencies (n (%)) in the two years prior to CKD index date, while complications were described as frequencies and rates (per 100 person-years) of events occurring following the CKD index date. Complications were described as acute (rates based on the number of events occurring after the CKD index date) and chronic (rates based on the time to the first occurrence of the event after the CKD index date). Stratifications by CKD stage [1–5], ASCVD, and T2DM status at index were assessed. All statistical analyses presented here were performed in SAS^®^ version 9.4.

## Results

**Fig. 1:**
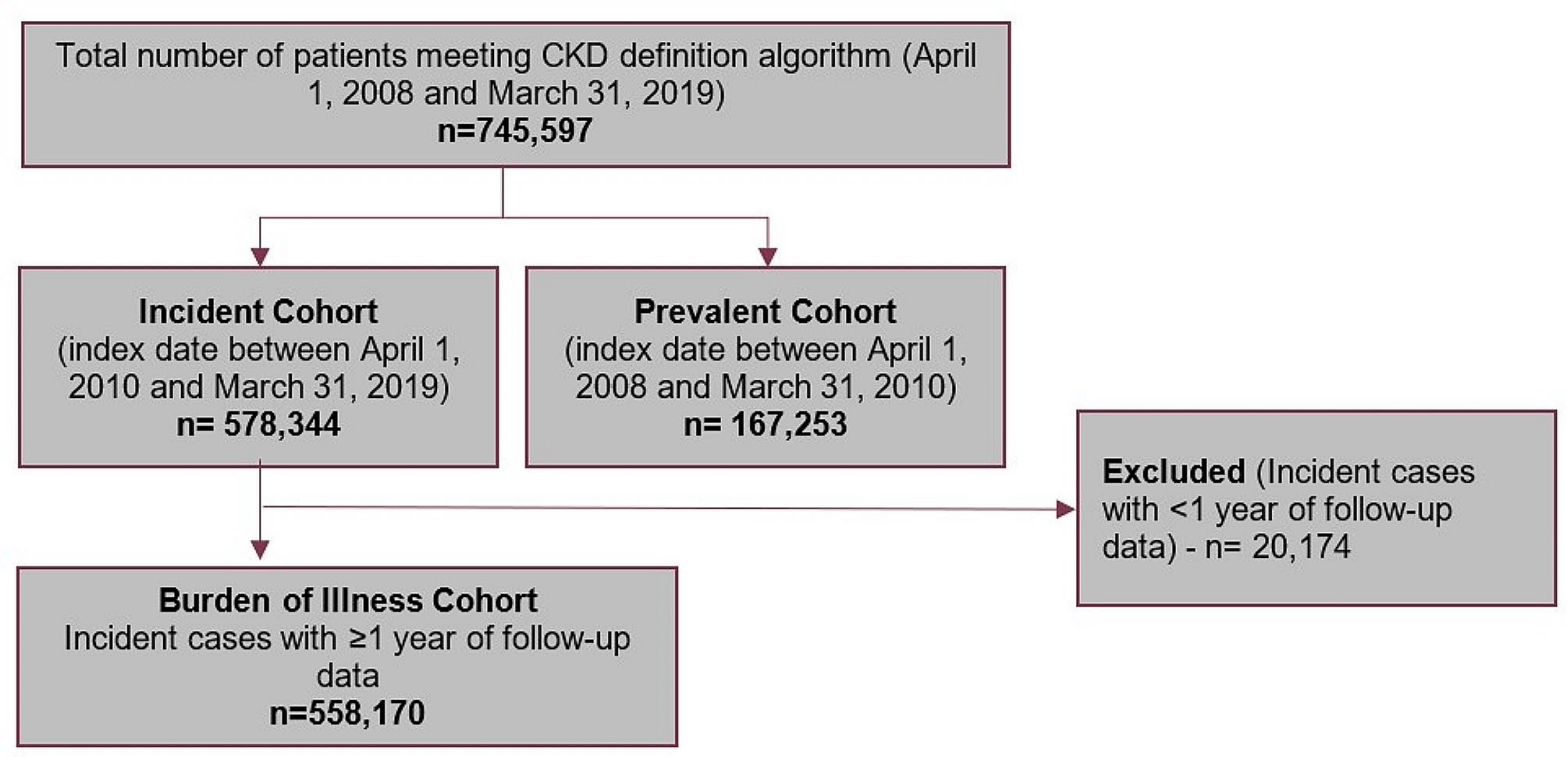
Flow chart for the CKD study cohort. Abbreviations: CKD: chronic kidney disease; eGFR: estimated glomerular filtration rate

**Fig. 2:**
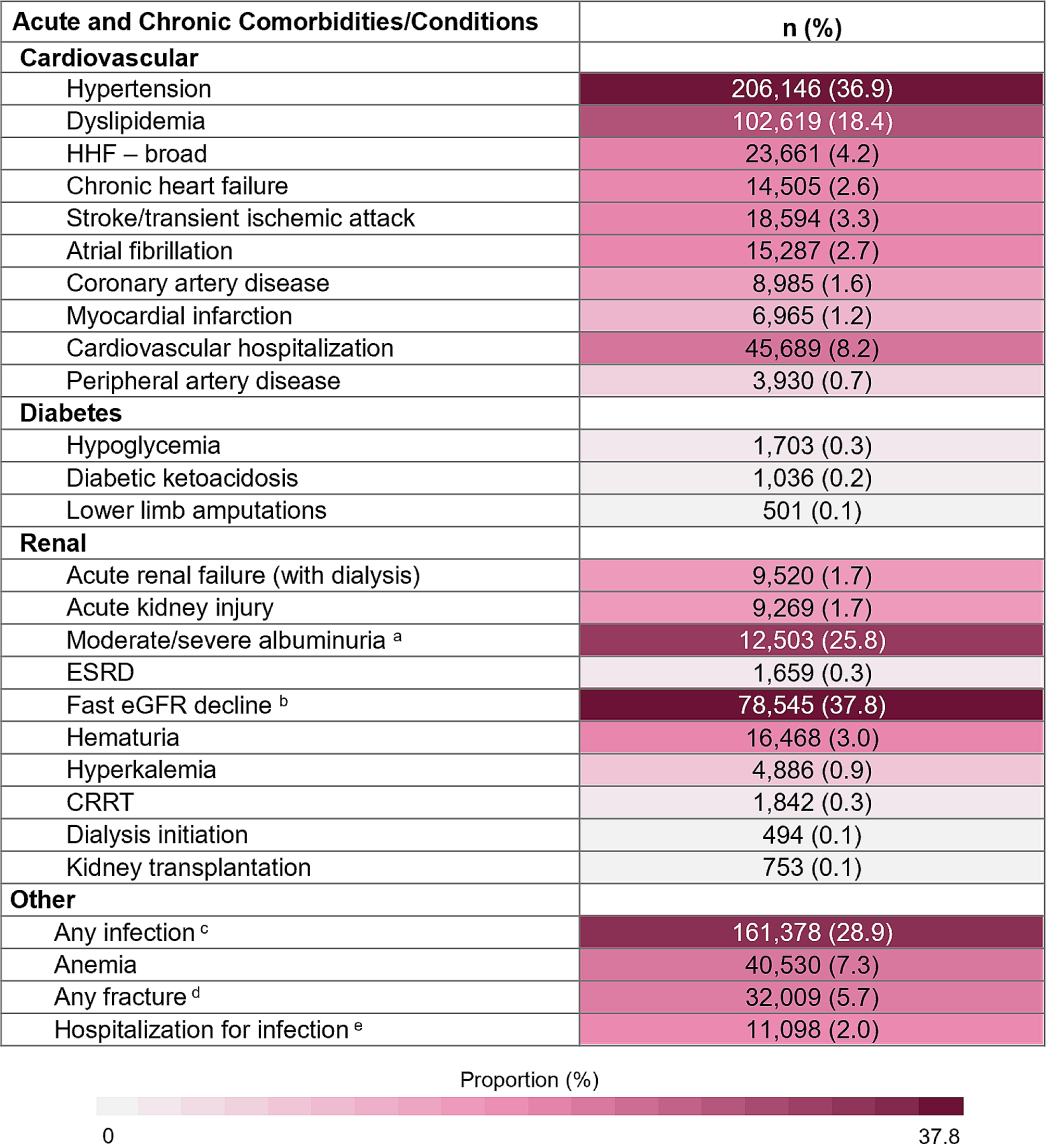
Acute/chronic comorbidities in two years prior to index of incident CKD cohort (*N* = 558,170). Abbreviations: CI: confidence interval; CRRT: continuous renal replacement therapy; eGRF: estimated glomerular filtration rate; ESRD: end stage renal disease; HHF: hospitalization for heart failure. ^a^ Due to the availability of laboratory data, the N-value for albuminuria was 48,391. ^b^ Due to the availability of laboratory data, the N-value for Fast eGFR decline was 207,565. ^c^ Any infection included: bacteremia, cellulitis abscess, gangrene, pneumonia, ulcer before gangrene, and urogenital tract infection. ^d^ Any fracture included: ankle, femur, foot, hand, hip, humerus, pelvis, radius, tibia, and upper limb. ^e^ Hospitalization for infection and death from infection included: bacteremia, cellulitis abscess, gangrene, pneumonia, and ulcer before gangrene

Overall, 588,170 patients with newly identified CKD and at least one year of follow-up data were included in our analyses (Fig. 1). Of the patients identified, 92% were identified using laboratory data. The mean ± SD of age for the CKD study cohort was 60.7 ± 15.3 years and half (50.3%) were male (Table 1). The mean (SD) follow-up time observed was 5.38 (2.20) years. Most patients were unable to be staged (79.9%) due to a lack of available laboratory data (albuminuria), and of patients that were staged, 14.3% were stage 3a. Most patients with CKD had a CCI score of 0 (68.9%), no ASCVD events/procedures in the two years prior to index (88.6%), and without T2DM in the two years prior to index (75.9%). The most frequently identified chronic comorbidities in the two years prior to CKD index date were hypertension (36.9%), and dyslipidemia (18.4%) (Fig. 2). Further, 11.4% and 2.6% had ASCVD and chronic heart failure, respectively.

**Table 1:** Patient characteristics for the CKD study cohort, stratified by CKD stage

Characteristics	Overall	No Stage	Stage 1	Stage 2	Stage 3a	Stage 3b	Stage 4	Stage 5
**Patients**,** n (%)**	558,170	446,010 (79.9)	3,739 (0.7)	11,706 (2.1)	80,095 (14.3)	14,770 (2.6)	1,775 (0.3)	75 (0.0)
**Age**,** n (%)**								
< 45	80,084 (14.3)	74,904 (16.8)	1,580 (42.3)	904 (7.7)	2,294 (2.9)	316 (2.1)	77 (4.3)	9 (12.0)
45-<55	96,208 (17.2)	86,299 (19.3)	999 (26.7)	1,930 (16.5)	6,291 (7.9)	587 (4.0)	85 (4.8)	17 (22.7)
55-<65	148,590 (26.6)	127,166 (28.5)	850 (22.7)	3,607 (30.8)	15,121 (18.9)	1,630 (11.0)	210 (11.8)	6 (8.0)
65-<75	131,567 (23.6)	100,238 (22.5)	290 (7.8)	3,503 (29.9)	23,908 (29.8)	3,315 (22.4)	299 (16.8)	14 (18.7)
≥ 75	101,721 (18.2)	57,403 (12.9)	20 (0.5)	1,762 (15.1)	32,481 (40.6)	8,922 (60.4)	1,104 (62.2)	29 (38.7)
Mean (SD)	60.7 (15.3)	58.5 (14.8)	46.9 (13.0)	62.3 (12.1)	70.4 (12.4)	75.9 (14.6)	75.7 (14.7)	66.9 (18.4)
**Sex**,** n (%)**								
Female	277,578 (49.7)	217,573 (48.8)	1,787 (47.8)	4,246 (36.3)	43,981 (54.9)	8,859 (60.0)	1,091 (61.5)	41 (54.7)
Male	280,592 (50.3)	228,437 (51.2)	1,952 (52.2)	7,460 (63.7)	36,114 (45.1)	5,911 (40.0)	684 (38.5)	34 (45.3)
**Geographic Region**,** n (%)**								
Calgary zone	215,195 (38.7)	166,875 (37.6)	924 (24.8)	3,906 (33.4)	35,529 (44.5)	7,037 (47.8)	895 (51.0)	29 (40.3)
Central zone	62,198 (11.2)	50,064 (11.3)	240 (6.4)	946 (8.1)	9,124 (11.4)	1,593 (10.8)	217 (12.4)	14 (19.4)
Edmonton zone	186,965 (33.6)	152,572 (34.3)	1,644 (44.1)	5,177 (44.3)	23,712 (29.7)	3,499 (23.8)	343 (19.6)	18 (25.0)
North zone	53,542 (9.6)	45,035 (10.1)	736 (19.7)	1,035 (8.8)	5,459 (6.8)	1,124 (7.6)	147 (8.4)	6 (8.3)
South zone	38,268 (6.9)	29,733 (6.7)	185 (5.0)	632 (5.4)	6,105 (7.6)	1,456 (9.9)	152 (8.7)	5 (6.9)
**CCI score**^**a**^, **n (%)**								
0	382,874 (68.9)	313,362 (70.6)	2,588 (69.4)	8,172 (70.1)	49,680 (62.3)	8,096 (55.1)	938 (53.6)	38 (53.5)
1–2	86,085 (15.5)	67,722 (15.3)	698 (18.7)	1,772 (15.2)	12,924 (16.2)	2,607 (17.7)	339 (19.4)	23 (32.4)
3+	86,691 (15.6)	62,928 (14.2)	441 (11.8)	1,706 (14.6)	17,140 (21.5)	3,992 (27.2)	474 (27.1)	10 (14.1)
Mean (SD)	1.0 (2.0)	0.9 (1.9)	0.8 (1.7)	0.9 (1.9)	1.4 (2.2)	1.7 (2.5)	1.7 (2.5)	1.1 (1.7)
**ASCVD at baseline**,** n (%)**								
No	494,384 (88.6)	399,665 (89.6)	3,422 (91.5)	10,046 (85.8)	67,560 (84.3)	12,133 (82.1)	1,491 (84.0)	67 (89.3)
Yes	63,786 (11.4)	46,345 (10.4)	317 (8.5)	1,660 (14.2)	12,535 (15.7)	2,637 (17.9)	284 (16.0)	8 (10.7)
**T2DM at baseline**,** n (%)**								
No	423,731 (75.9)	345,687 (77.5)	1,448 (38.7)	2,585 (22.1)	61,757 (77.1)	10,960 (74.2)	1,241 (69.9)	53 (70.7)
Yes	134,439 (24.1)	100,323 (22.5)	2,291 (61.3)	9,121 (77.9)	18,338 (22.9)	3,810 (25.8)	534 (30.1)	22 (29.3)

Abbreviations: ACR: albumin-to-creatinine ratio, ASCVD: atherosclerotic cardiovascular disease; CCI: Charlson Comorbidity Index; HbA1c: Hemoglobin A1c; SD: standard deviation; T2DM: type 2 diabetes mellitus

^a^ The Charlson comorbidity index score was derived based on hospitalizations (DAD) and physician visits in the one year prior to the CKD index date

^b^ Comorbidities of interest and treatments were identified in the two-years prior to the CKD index date

**Fig. 3:**
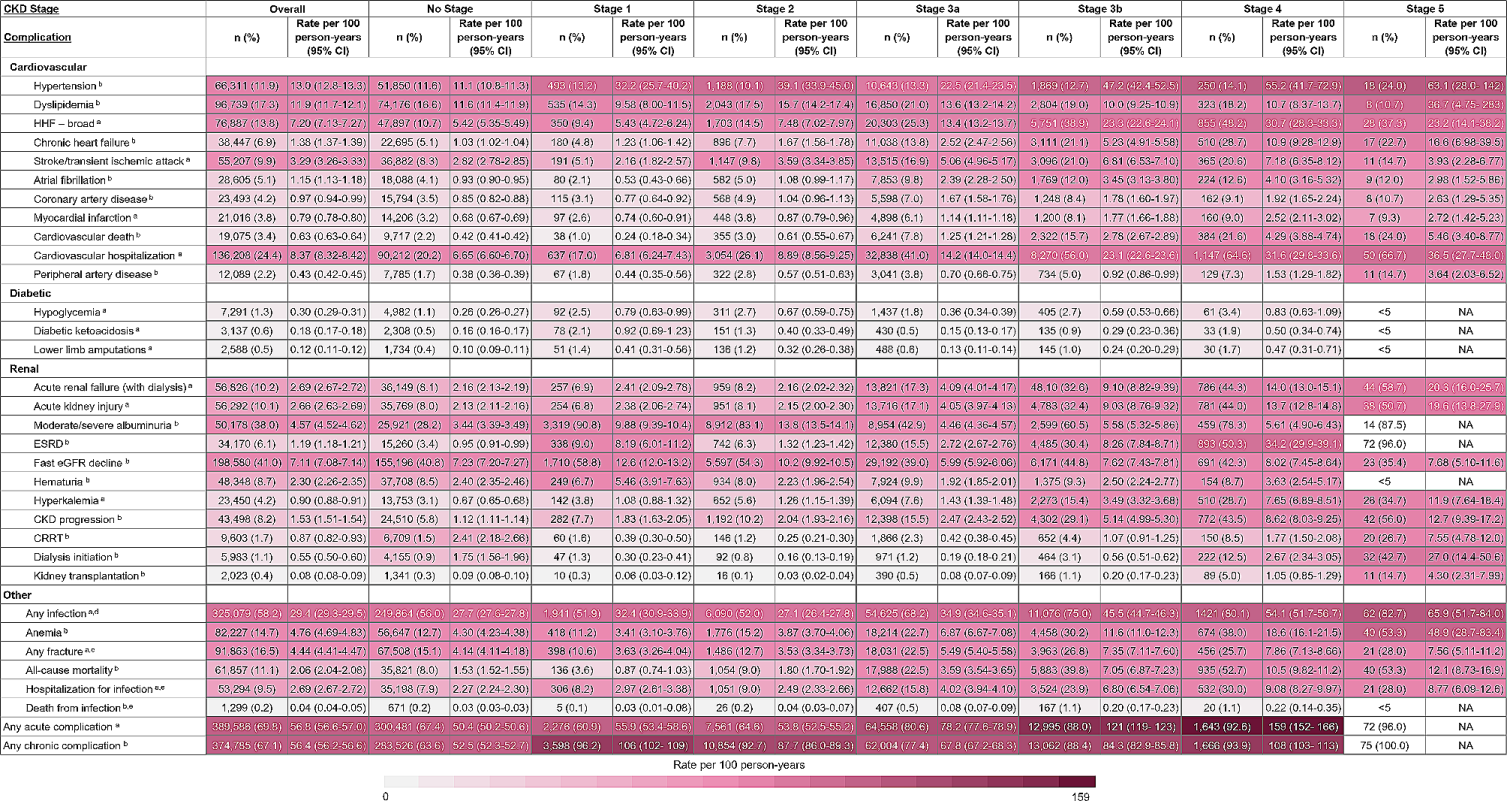
Complication profiles/rates in incident CKD cohort by stage (index to March 31, 2020). Abbreviations: CI: confidence interval; CRRT: continuous renal replacement therapy; eGRF: estimated glomerular filtration rate; ESRD: end stage renal disease; HHF: hospitalization for heart failure. ^a^ Acute complication rates were calculated based on the number of events that occurred after the CKD index date (rate of complication by year). ^b^ Chronic complication rates were calculated based on the time to the first occurrence of the event after the CKD index date. ^c^ Any fracture included: ankle, femur, foot, hand, hip, humerus, pelvis, radius, tibia, and upper limb. ^d^ Any infection included: bacteremia, cellulitis abscess, gangrene, pneumonia, ulcer before gangrene, and urogenital tract infection. ^e^ Hospitalization for infection and death from infection included: bacteremia, cellulitis abscess, gangrene, pneumonia, and ulcer before gangrene. NA – Rate per 100 person-years is not available due to small sample sizes

Following CKD index date, the most frequently identified acute complications were recurrent infection (*n* = 325,079; 58.2%) and hospitalization for cardiovascular disease (*n* = 136,208; 24.4%), corresponding to rates (95% confidence interval [CI]) of 29.4 (29.3–29.5) and 8.37 (8.32–8.42) per 100 person-years, respectively (Fig. 3). The most common chronic complications were dyslipidemia (*n* = 96,739; 17.3%), anemia (*n* = 82,227; 14.7%), and hypertension (*n* = 66,311; 11.1%), corresponding to rates (95% CI) of 11.9 (11.7–12.1), 4.76 (4.69–4.83), and 13.0 (12.8–13.3) per 100 person-years, respectively. Overall, those who had higher stages of CKD at index tended to have higher rates of complications throughout follow-up. For example, individuals with stage 1 CKD at index had an infection rate (95% CI) of 32.4 (30.9–33.9) per 100 person-years, compared to individuals with stage 5 CKD at index who had an infection rate of 65.9 (51.7–84.0) per 100 person-years. Moreover, hypertension occurred in individuals with stage 1 CKD at index at a rate 32.2 (25.7–40.2) per 100 person-years, compared to 63.1 (28.0- 142) in individuals with stage 5 CKD at index.

**Fig. 4:**
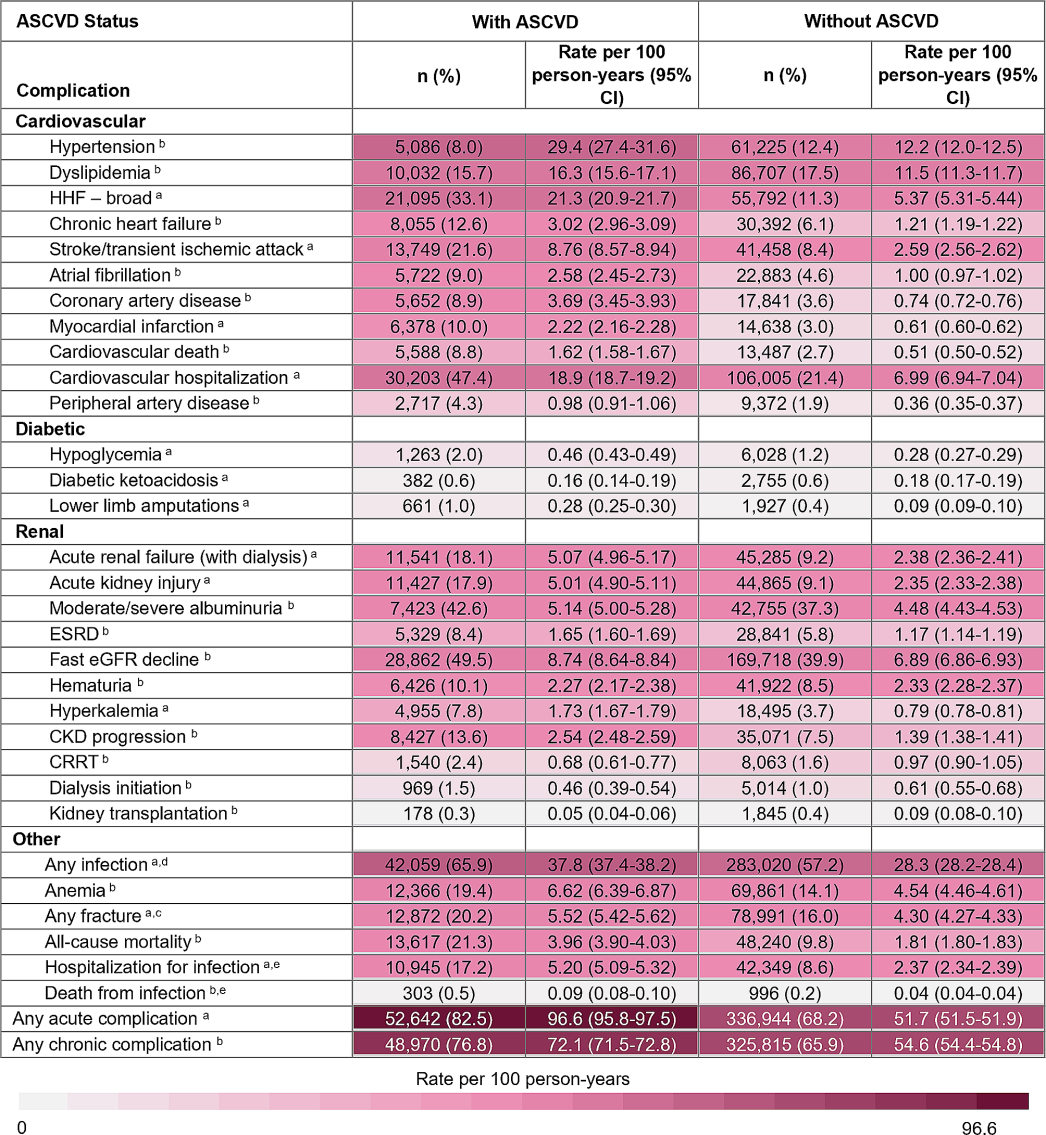
Complication profiles/rates in incident CKD cohort, stratified by ASCVD status at index. Abbreviations: CI: confidence interval; CRRT: continuous renal replacement therapy; eGRF: estimated glomerular filtration rate; ESRD: end stage renal disease; HHF: hospitalization for heart failure. ^a^ Acute complication rates were calculated based on the number of events that occurred after the CKD index date (rate of complication by year). ^b^ Chronic complication rates were calculated based on the time to the first occurrence of the event after the CKD index date. ^c^ Any fracture included: ankle, femur, foot, hand, hip, humerus, pelvis, radius, tibia, and upper limb. ^d^ Any infection included: bacteremia, cellulitis abscess, gangrene, pneumonia, ulcer before gangrene, and urogenital tract infection. ^e^ Hospitalization for infection and death from infection included: bacteremia, cellulitis abscess, gangrene, pneumonia, and ulcer before gangrene

Among those who had an ASCVD event/procedure two years prior to index, the proportions and rates of complications following CKD index date were generally higher, relative to those without an ASCVD event/procedure (Fig. 4). For example, individuals with ASCVD at index had an infection rate (95% CI) of 37.8 (37.4–38.2), per 100 person-years compared to individuals without ASCVD at index who had an infection rate of 28.3 (28.2–28.4) per 100 person-years. However, diabetic ketoacidosis, hematuria, continuous renal replacement therapy (CRRT), dialysis initiation, and kidney transplantation had both slightly higher proportions and rates amongst those without an ASCVD event/procedure, compared to those with ASCVD.

**Fig. 5:**
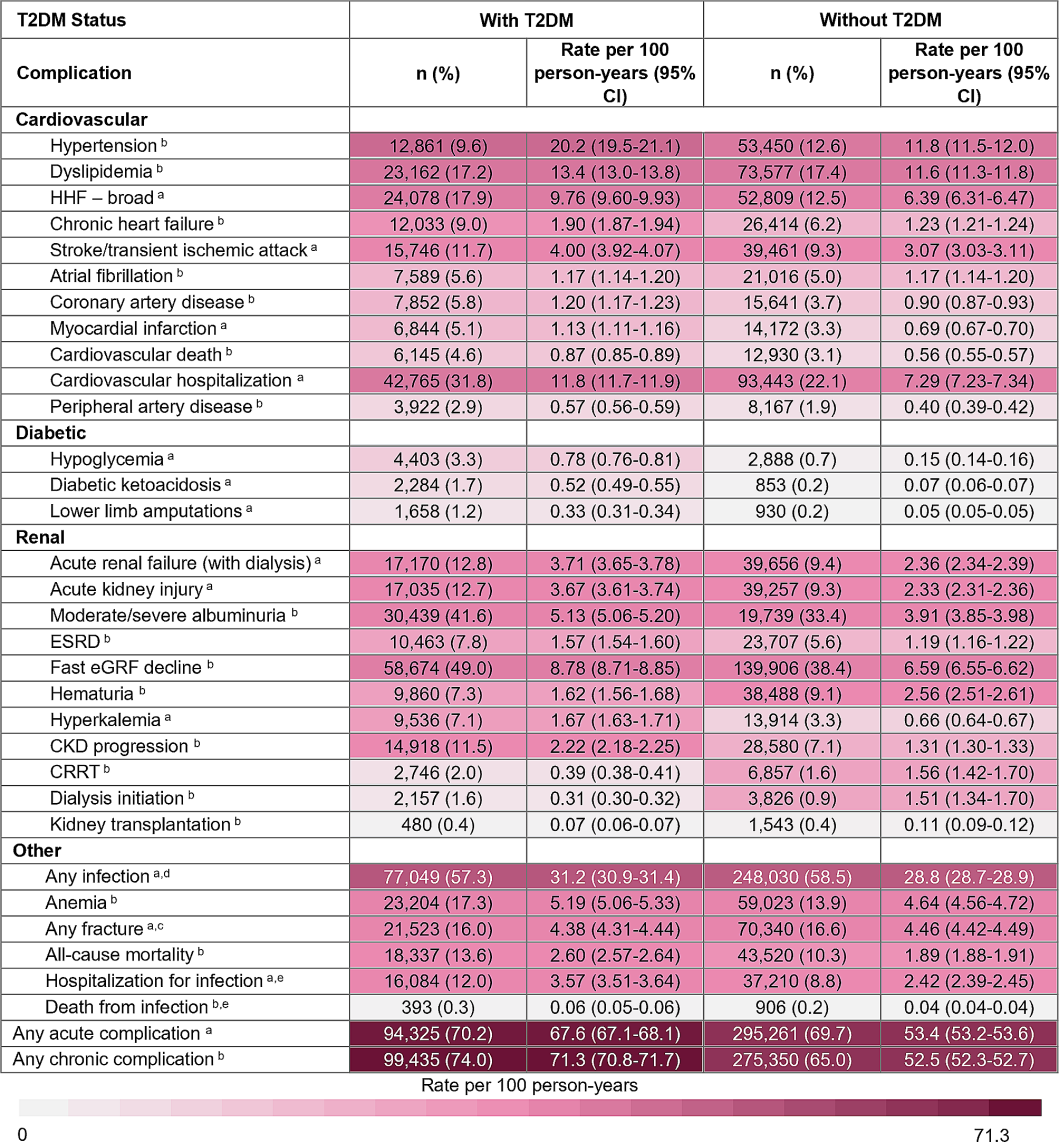
Complication profiles/rates in incident CKD cohort, stratified by T2DM status at baseline. Abbreviations: CI: confidence interval; CRRT: continuous renal replacement therapy; eGRF: estimated glomerular filtration rate; ESRD: end stage renal disease; HHF: hospitalization for heart failure. ^a^ Acute complication rates were calculated based on the number of events that occurred after the CKD index date (rate of complication by year). ^b^ Chronic complication rates were calculated based on the time to the first occurrence of the event after the CKD index date. ^c^ Any fracture included: ankle, femur, foot, hand, hip, humerus, pelvis, radius, tibia, and upper limb. ^d^ Any infection included: bacteremia, cellulitis abscess, gangrene, pneumonia, ulcer before gangrene, and urogenital tract infection. ^e^ Hospitalization for infection and death from infection included: bacteremia, cellulitis abscess, gangrene, pneumonia, and ulcer before gangrene

When cardiovascular, diabetic, renal, and other complications were stratified by T2DM. status at index, most complications rates were higher in those with T2DM at index (Fig. 5). However, exceptions to this trend were observed, specifically among renal complications and fractures where complication rates were lower. Like the results observed among ASCVD stratifications, individuals without T2DM at index had slightly higher proportions and rates for diabetic ketoacidosis, hematuria, CRRT, dialysis initiation, and kidney transplantation. Moreover, individuals without T2DM at baseline who experienced a fracture (16.6%), did so at a slightly higher rate (4.46 (4.42–4.49) per 100 person-years) than individuals with T2DM at baseline (16.0%; 4.38 (4.31–4.44) per 100 person-years).

## Discussion

In this study using Alberta administrative healthcare data, patients with a new diagnosis of CKD were identified. Among patients with CKD, cardiovascular comorbidities and complications including, hypertension and dyslipidemia, as well as infections were commonly reported across the follow-up period. Among stratified analyses, those with more advanced CKD, an ASCVD event/procedure, or T2DM in the two years prior to their CKD index date, had higher rates of complications, although exceptions to this trend were observed. To our knowledge, this is the first study in Canada to comprehensively evaluate a wide range of medical complications prior to and following CKD identification. This study demonstrates notable estimates of several major comorbidities and complications in patients with CKD from real-world clinical settings.

The comorbidity profiles in this cohort were generally consistent with those of other published literature. A Canadian-based population survey study reported hypertension and T2DM comorbidity rates in CKD patients at 33.6% and 19.6% respectively, relative to the 36.9% and 24.1% reported for all CKD stages in this study, noting significant variations among study designs [1]. Based on these results, systemic screening of hypertensive patients for CKD might be considered from a healthcare or guideline implementation perspective.

The subsequent complication rates in patients with newly identified CKD were consistent with trends reported within the literature, increasing with respect to CKD staging [4–6]. There is a documented correlation between the prevalence of anemia and CKD severity [30]; a previous study reported a 25% prevalence of anemia in stage 1 patients and 50% among those with stage 2 through 4. Our study revealed that anemia occurred in 11.2%, 15.2%, 22.7%, 30.2%, 38.0%, and 53.3% in CKD stages 1, 2, 3a, 3b, 4, and 5, respectively. The lower estimates of anemia in early stages of CKD reported in our study compared to those reported in other studies can likely be attributed to variations in study methods (i.e., algorithmic approach) used to define the study populations as well as, the availability of laboratory data. While prevalence of infection has been previously studied among this population, [31] the definitions and types of infections investigated here do not allow for direct comparisons. However, CKD has been associated with higher risk of bacterial and viral respiratory tract infections, which were also prevalent among patients in this study, as observed by high rates of infection.

The higher complication rates among subgroups of patients with ASCVD and T2DM provide further evidence that cardiovascular outcomes are prevalent in CKD and are likely a leading cause of morbidity and mortality [32, 33]. Further, our results suggest that the combination of comorbidities from the cardio-renal-metabolic spectrum increase the rate of complications and worsen the complication profiles vs. patients without the comorbidities. Of note, higher rates (per 100 person-years) of hypertension and dyslipidemia were observed in patients with ASCVD, although the proportions of patients with these conditions captured after the CKD index date were higher in patients without ASCVD at baseline. This difference could be due the limitations of the methodology (further described below), where patients with hypertension and dyslipidemia prior to the CKD index date where not excluded from the analyses and it is likely that these conditions were already present in patients with ASCVD at the CKD index date and not captured again after. The higher rates of hypertension and dyslipidemia in patients with ASCVD are likely due to these outcomes being captured earlier in the follow-up period from the CKD index date (as these are likely comorbidities of ASCVD).

This study was a large, population-based sample of adult patients with CKD identified using validated algorithms that combined diagnostic coding and laboratory values in Alberta, Canada. Results from our study provide prevalence and rate estimates detailed by CKD stage and comorbidity categorizations from real-world clinical settings. While other published literature has focused largely on renal and cardiovascular outcomes, our study adopted a more comprehensive approach, detailing included infections and fracture rates in patients with CKD. Finally, stratifying the complications by ASCVD and T2DM status at index provided evidence of subpopulations that are most affected by CKD-related complications.

Limitations should be noted when interpreting these results. The CKD index date was anticipated to approximate the patients’ diagnosis date, but due to the timeframe of the data and algorithm used, patients may have been diagnosed at an earlier date than indexed. Thus, the term ‘newly identified’ was used instead of ‘newly diagnosed’. While there was a small proportion of patients excluded from the burden of illness cohort due to having < 1 year of follow-up data, including these patients could have introduced survivorship bias. Further, administrative data is not collected specifically for research, but rather for billing, monitoring, and hospital administrative purposes. Potential misclassification bias of the CKD cohort and comorbidity/complication outcome definitions due to incorrect/unavailable coding or laboratory values may be present. Since conditions identified prior to the CKD index date were not excluded in the complications analyses, this may have overestimated the rates of complications; however, excluding the conditions present prior to CKD index date would underestimate the complications since they are dependent on how events were captured in the administrative data. Lastly, a large proportion of our cohort did not have albuminuria testing to define stage 1–2 CKD. Therefore, most patients could not be staged at their CKD index date. Only 48,391/558,170 patients (8.6%) had an albuminuria test available before their CKD index date, meaning most patients were identified based on eGFR or diagnostic coding criteria.

While administrative data is valuable in collecting population-level data, it does not collect other important patient characteristics such as ethnicity, lifestyle factors, and behaviors. Various patient and clinical characteristics may represent high-risk groups or determinants of health disparities in CKD diagnosis and management. Of particular interest, socioeconomic status which intersects with race/ethnicity, has been shown to be a determinant of health disparities in CKD [34, 35]. The risk among the poor is associated with their greater burden of clinical risk factors including, albuminuria, diabetes, and hypertension [35]. Moreover, food insecurity has been established as an important factor, as income relates to diet quality [36]. Future research utilizing a more comprehensive CKD database with physician confirmed CKD diagnoses, that includes information beyond what is captured for health administration, such as income would offer insights into different high-risk subgroups and health disparities [37]. 

## Conclusion

CKD affects a substantial proportion of the adult population in Alberta. The burden of CKD is further enhanced by comorbidities, and high rates of complications, especially cardiovascular-related complications, such as hypertension and dyslipidemia, which often lead to more serious events or outcomes. Determining better strategies to implement optimal care for patients living with CKD is needed, including early detection and improved management of CKD including subsequent complications.

### Electronic supplementary material

Below is the link to the electronic supplementary material.

**Additional file 1**: Supplementary files including administrative data sources used, algorithms, disease stages, ASCVD definitions, and disease complications.

## Data Availability

Data were obtained via data request to Alberta Health and Alberta Precision Laboratories. Data from this study are not publicly available and cannot be shared due to privacy reasons and ethical restrictions, as per the research agreement with Alberta Health and Alberta Precision Laboratories.

## Abbreviations

ACR: Albumin: creatinine ratio
ASCVD: Atherosclerotic cardiovascular disease
CCI: Charlson Comorbidity Index
CKD: Chronic kidney disease
eGFR: Estimated glomerular filtration rate
HbA1c: Hemoglobin A1C
SD: Standard deviation
T2DM: Type 2 diabetes mellitus
