# Supplementary material for: Acute and chronic complication profiles among patients with chronic kidney disease in Alberta, Canada: a retrospective observational study
Source: BMC Nephrol. 2024 Jul 29;25:244. doi: 10.1186/s12882-024-03682-z (PMC11288078; doi:10.1186/s12882-024-03682-z)
Supplement: Supplementary file 1 — Additional file 1: Supplementary files including administrative data sources used, algorithms, disease stages, ASCVD definitions, and disease complications. [file 12882_2024_3682_MOESM1_ESM.docx]

**Supplementary Materials**

**Supplementary Table 1. Administrative data sources used**

| **Dataset** | **Description*** |
| --- | --- |
| Alberta Blue Cross Pharmacy Claims | Health benefit data primarily on seniors and their dependents |
| Ambulatory Care – National Ambulatory Care Reporting System | Includes data from all emergency department-based and community-based ambulatory care, including information on services, diagnostic and procedure codes |
| Inpatient Hospitalizations – Discharge Abstract Database | Includes data from inpatient stays, including information on services, diagnostic and procedure intervention codes as well as length of stay |
| Pharmaceutical Information Network Dispenses | Includes information on medication dispenses and associated information at the pharmacy level (all private and public plans) |
| Population Registry | Includes basic demographic information, including age, gender, and zone |
| Practitioner Claims | Includes provider claims data for physicians and other providers for insured health services, and reports on provider and service data |
| Vital statistics – deaths | Includes death information; need authorization from Service Alberta |

*Taken from the Alberta Health, Analytics and Performance Reporting Branch – Overview of Administrative Health Datasets, April 28, 2017 - ^©^2017 Government of Alberta

**Supplementary Table 2. Validated algorithm for case definition of CKD study cohort**

| **Diagnosis Condition** | **ICD-9-CM** | **ICD-10-CA** | **Definition Algorithm** |
| --- | --- | --- | --- |
| Chronic kidney disease | 583, 584, 585, 586, 592, 593.9 | N00-N23 | At least 1 IP **or**  3 claims within 1 year **or** mean eGFR <90 mL/min*1.73m^2^ **or**  mean albuminuria ≥3 mg/mmol over 12 months^a^ |

Abbreviations: eGFR: estimated glomerular filtration rate; ICD 9-CM: The International Classification of Diseases, Ninth Revision, Clinical Modification; ICD-10-CA: International Statistical Classification of Diseases and Related Health Problems, 10^th^ Revision, Canada; IP: inpatient.

^a^The mean eGFR or albuminuria is based on two consecutive tests at least 90 days apart and within 12 months.

**Supplementary Table 3. 2012 KDIGO CKD stages**

| **CKD Stage** | **2012 KDIGO Guideline Definition** |
| --- | --- |
| Stage 1 | > 90 mL/min/1.73 m^2^ eGFR level with moderate or greater albuminuria (see **Supplementary Table 3**) |
| Stage 2 | 60 – 89 mL/min/1.73 m^2^ eGFR level with moderate or greater albuminuria (see **Supplementary Table 3**) |
| Stage 3a | 45 – 59 mL/min/1.73 m^2^ eGFR level |
| Stage 3b | 30 – 44 mL/min/1.73 m^2^ eGFR level |
| Stage 4 | 15 – 29 mL/min/1.73 m^2^ eGFR level |
| Stage 5 | < 15 mL/min/1.73 m^2^ eGFR level |

Abbreviations: CKD: chronic kidney disease; eGFR: estimated glomerular filtration rate; KDIGO: Kidney Disease Improving Global Outcomes

Note: these stages may be updated according to future KDIGO guideline updates.

**Supplementary Table 4. 2012 KDIGO albuminuria stages**

| **Albuminuria Stage** | **2012 KDIGO Guideline Definition** |
| --- | --- |
| None/mild | ACR < 3 mg/mmol, PCR < 15 mg/mmol |
| Moderate | ACR 3 – 30 mg/mmol, PCR 15-50 mg/mmol |
| Severe | ACR > 30 mg/mmol, PCR > 50 mg/mmol |

Abbreviations: ACR: albumin:creatinine ratio; KDIGO: Kidney Disease Improving Global Outcomes; PCR: protein:creatinine ratio

**Supplementary Table 5. ASCVD definitions – diagnostic and procedure codes**

| **ASCVD condition** | **ICD-9-CM** | **ICD-10-CA** | **Algorithm Definition** |
| --- | --- | --- | --- |
| Acute myocardial infarction | 410 | I21, I22 | 1 IP (Inpatient only), any position (discharge date as index date) |
| Stable/unstable angina | 411, 413 | I20 | 1 IP (inpatient only), any position; OR 1 practitioner claims any position; OR 1 ED visit, any position (all discharge date=index date); earliest as index date |
| Cerebrovascular disease/stroke | 430-434, 436-438, 3623 | I60-I65, I67, I69, H341 | 1 IP (discharge date); OR 2 practitioner claims/ED visits at least 30 days apart; any position (2nd date=index date) earliest as index date |
| Transient ischemic attack | 435 | G450-G453, G458-G459, H340 | 1 IP, any position (discharge date=index date); OR 2 practitioner claims/ED visits 30 days apart, any position (2nd date=index date); earliest as index date |
| Coronary atherosclerosis/old myocardial infarction | 412, 414 | I25 | 1 IP (discharge date, any position); OR 2 practitioner claims/ED visits (2nd date=index date), claims at least 30 days apart; any position; earliest as the index date |
| Peripheral arterial disease | 4439, 4402 | I739, I702, I792 | 1 IP (any position, discharge date); OR 2 practitioner claims/ED visits30 days apart (2nd date=index date); earliest as index date |
| Percutaneous coronary intervention | CCP codes: 4802, 4803 | CCI codes: 1IJ50, 1IJ57GQ, 1IJ54 | 1 IP or 1 ED CCP/CCI code (any position, discharge date); earliest date as the index date |
| Coronary artery bypass graft surgery | CCP codes: 481 | CCI codes: 1IJ76 | 1 IP or 1 ED CCP/CCI code (any position, discharge date earliest date as the index date |

Abbreviations: CCI: Canadian Classification of Health Intervention; CCP: Canadian Classification of Diagnostic, Therapeutic, and Surgical Procedures; ED: emergency department; ICD 9-CM: The International Classification of Diseases, Ninth Revision, Clinical Modification; ICD-10-CA: International Statistical Classification of Diseases and Related Health Problems, 10th Revision; IP: inpatient.

**Supplementary Table 6. T2DM definition and diagnostic codes**

| **Diagnosis Condition** | **ICD-9-CM** | **ICD-10-CA** | **Definition Algorithm** |
| --- | --- | --- | --- |
| Type 2 diabetes mellitus | 250 | E11-E14 | Presence of at least one ICD-9-CM/ICD-10-CA code (from DAD, NACRS, or Practitioner Claims) |

Abbreviations: DAD: discharge abstract database; ICD 9-CM: The International Classification of Diseases, Ninth Revision, Clinical Modification; ICD-10-CA: International Statistical Classification of Diseases and Related Health Problems, 10^th^ Revision, Canada; NACRS: National Ambulatory Care Reporting System.

**Supplementary Table 7. Complication outcome definitions – diagnostic and procedure codes**

| **Complication** | **ICD/Procedure Codes** | **Definition** |
| --- | --- | --- |
| **Cardiovascular** |  |  |
| Hypertension | ICD-9-CM: 401-405  ICD-10-CA: I10-I13, I15 | 1 IP or 2 claims in 2 years or less |
| Dyslipidemia | ICD-9-CM: 272  ICD-10-CA: E78 | 1 IP, 1 ED or 1 claim |
| HHF – broad | ICD-9-CM: 402.0, 402.1, 402.9, 404.0, 404.1, 404.9, 425.4-425.9, 428  ICD-10-CA: I09.9, I25.5, I42.0, I42.5–I42.9, I43, I50 | 1 IP, 1 ED or 1 claim |
| Chronic heart failure | ICD-9-CM: 402.0, 402.1, 402.9, 404.0, 404.1, 404.9, 425.4-425.9, 428  ICD-10-CA: I09.9, I25.5, I42.0, I42.5–I42.9, I43, I50 | 1 IP or 2 claims in 2 years or less |
| Stroke/transient ischemic attack | ICD-9-CM: 362.3, 430, 431, 433.0-3, 433.8-9, 434.0-1, 434.9, 435, 436  ICD-10-CA: G45.0-G45.3, G45.8, G45.9, H34.1, I60, I61, I63, I64 | 1 IP, 1 ED or 1 claim |
| Atrial fibrillation | ICD-9-CM: 427.3  ICD-10-CA: I48.0 | 1 IP or 2 claims in 2 years or less |
| Coronary artery disease | See Supplementary Table 3 for ICD/procedure codes. | 1 IP for myocardial infarction, coronary artery bypass graft surgery, or percutaneous coronary intervention) |
| Myocardial infarction | ICD-9-CM: 410  ICD-10-CA: I21-I22 | 1 IP |
| Cardiovascular death | ICD-9-CM: 410-414, 430-438, 390-398, 401-405, 410-417, 420-438, 440-444, 446-448, 451-459  ICD-10-CA: I21-I25, I60-I69, I00-I21, !26-I59, I70-I199 | Any of the following:   - 1 IP record with discharge disposition as death AND relevant ICD codes - 1 ED record with disposition as death AND relevant ICD codes - 1 vital statistics record with relevant ICD codes |
| Peripheral artery disease | ICD-9-CM: 440.2  ICD-10-CA: I70.2 | 1 IP, 1 ED or 1 claim |
| Cardiovascular hospitalization | See all individual cardiovascular outcomes listed. | 1 IP from any of the above cardiovascular events |
| **Diabetic** |  |  |
| Hypoglycemia | ICD-9-CM: 251.0, 251.2, 251.4, 251.5, 251.8, 251.9  ICD-10-CA: E15, E16 | 1 IP or 1 claim |
| Diabetic ketoacidosis | ICD-9-CM: 250.10, 250.11, 250.1, 250.19  ICD-10-CA: E10.1, E11.1, E13.1, E14.1 | 1 IP |
| Lower limb amputations | ICD-10-CA: S53.43-S53.49  CCP: 96.12, 96.14, 96.15, 96.16, 96.17, 96.18  CCI: 1.VA.93.^^, 1.VC.93.^^, 1.VG.93.^^, 1.VQ.93.^^, 1.WA.93.^^, 1.WE.93.^^, 1.WI.93.^^, 1.WJ.93.^^, 1.WK.93.^^, 1.WL.93.^^, 1.WM.93.^^, 1.WN.93.^^ | 1 IP or 1 claim |
| **Renal** |  |  |
| Acute renal failure (with dialysis) | ICD-9-CM: 584.5-9, V45.1, V56.0  ICD-10-CA: N17.0, N17.1, N17.2, N17.8, N17.9  CCI: 5.A1.D7.0Z, 5.A1.D8.0Z, 5.A1.D9.0Z, 5.A1.D0.0Z, 1.PZ.21.HQ-BS, 1.JQ.53, 1.JT.53 | 1 IP |
| Acute kidney injury | ICD-9-CM: 584.5-9  ICD-10-CA: N17.0, N17.1, N17.2, N17.8, N17.9 | 1 IP |
| Moderate/Severe albuminuria | Albuminuria was categorized as follows:  **None/mild** – ACR <3 mg/mmol, PCR <15 mg/mmol  **Moderate** – ACR 3-30 mg/mmol, PCR 15-50 mg/mmol  **Severe** – ACR >30 mg/mmol, PCR >50 mg/mmol | Using the participant’s most recent outpatient measurement within two years before the index date based on either the albumin:creatinine ratio (ACR), and the protein:creatinine ratio (PCR). A PCR assessment was used when ACR was not available.^22^ |
| ESRD | ICD-9-CM: 585, V56.0, V56.8, V45.1, V42.0  ICD-10-CA: N18.5-6, Z49.31, Z49.32, Z99.2, Z91.15, Z49.2, Z94.0, T86.10-T86.12  CCP: 51.95, 66.98, 51.27, 67.01, 67.11, 67.5, 71.8, 26.35, 83.9, 33.9, 67.51, 68.51, 70.0, 67.02  CCI: 1.PZ.21, 1.KY.54, 1.PE.57, 1.PC.56, 1.KY.76, 2.ZZ.13, 1.CG.76, 2.OZ.29, 7.SJ.32, 1.PC.83.LA, 1.PC.85 | Any of the following:   - ESRD, defined as two codes (2 records; IP, ED or claim), separated by at least 30 days - Kidney transplantation defined as 1 IP, 1 ED or 1 claim |
| Faster eGFR decline | A 5 unit (mL/min/1.73 m^2^) decrease in eGFR decline per year ^33^ | Based on laboratory values |
| Hematuria | ICD-9-CM: 599.7  ICD-10-CA: R31 | 1 IP, 1 ED or 1 claim |
| Hyperkalemia | ICD-9-CM: 276.7  ICD-10-CA: E87.5 | 1 IP, 1 ED or 1 claim |
| CKD Progression | Decline of 35% or greater in eGFR from baseline more than 3 months after the index date (baseline eGFR <60 mL/min/1.73 m^2^)^34^  GFR will be calculated based on the CKD-EPI calculation  GFR equation = 141 * min(Scr/κ,1)^α^ * max(Scr/κ, 1)^-1.209^ * 0.993^Age^ * 1.018 [if female]  Scr is serum creatinine (mg/dL)  κ is 0.7 for females and 0.9 for males  α is 0.329 for females and 0.411 for males  min indicates the minimum of Scr/κ or 1, and max indicates the maximum of Scr/κ or 1 | Based on laboratory values |
| CRRT | ICD-9-CM: V45.1, V56  ICD-10-CA: N17.0, N17.1, N17.2, N17.8, N19.9, N11.1, N13.1, N13.5  CCI: 5.A1.D7.0Z, 5.A1.D8.0Z, 5.A1.D9.0Z, 5.A1.D0.0Z, 1.PZ.21.HQ-BS, 1.JQ.53, 1.JT.53, 1.PQ.78.^^, 1.PQ.80.^^ | 1 IP, 1 ED or 1 claim |
| Dialysis initiation | ICD-9-CM: V45.1, V56  ICD-10-CA: Z99.2, Z49.0, Z49.1  CCI: 1.PZ.21.HQ-BR, 1.PZ.21.HQ-BS, 1.JQ.53, 1.JT.53 | 1 IP, 1 ED or 1 claim |
| Kidney transplantation | ICD-9-CM: V42.0  ICD-10-CA: Z94.0, T861.0-2  CCP: 67.5, 67.59, 67.59A  CCI: 1.PC.83.LA, 1.PC.85.^^ | 1 IP, 1 ED or 1 claim |
| **Other** |  |  |
| Anemia | ICD-9-CM: 280-285  ICD-10-CA: D50-D64 | 1 IP or 1 claim |
| Any infection | See ICD codes for individual infections listed below. | Any urogenital tract infections, pneumonia, bacteremia, cellulitis/abscess, gangrene, ulcer (before gangrene) |
| Hospitalization for infection | See ICD codes for individual infections listed below. | 1 IP claim for pneumonia, bacteremia, gangrene, or ulcer (before gangrene) |
| Death from infection | See ICD codes for individual infections listed below. | Death where cause of death is listed as pneumonia, bacteremia, cellulitis/abscess, gangrene, or ulcer (before gangrene) – taken from vital statistics |
| Urogenital tract infection | ICD-9-CM: 016.0-016.3, 025.0, 041.1, 041.3-4, 041.8-9, 078.5, 079.9, 095, 098.1-3, 099, 102.1, 128.9, 131.0, 131.8-9, 590.0-590.3, 590.8-9, 593.8, 595, 597.0, 597.8, 598.0x, 599, 054.1, 112.1-2, 601.x, 603.1, 604.0, 604.9, 607.0-1, 608.0, 608.4, 608.83, 614.x-616.x, 622.8, 624.8, 682.2, 995.91  ICD-10-CA: A18.1, A24.4, B95.8, B96.1, B96.2, B96.8, B25.9, B97.88, A52.7, A54.2, A56.1, A66.1, B83.9, A59.0, N11, N28.88, N30, N34.2, N35.1, N39.0, A60.9, B37.3, N41.0, N43.1, N45.1-N45.3, N48, N48.1, N49.0, N49.9, N50.1, N70-N72, N76, N88.8, N90.8, L03.3, A41.9 | 1 IP, 1 ED or 1 claim |
| Pneumonia | ICD-9-CM: 480-486  ICD-10-CA: J12-J18, J22, J95 | 1 IP, 1 ED or 1 claim |
| Cellulitis/abscess | ICD-9-CM: 681.0-1, 681.9, 682.0-682.9  ICD-10-CA: L03 | 1 IP, 1 ED or 1 claim |
| Ulcer (before gangrene) | ICD-9-CM: 707  ICD-10-CA: L89 | 1 IP, 1 ED or 1 claim |
| Gangrene | ICD-9-CM: 040.0, 440.2, 785.4  ICD-10-CA: A48.0, I70.21, N49.8, E11.51, E11.68, E13.68, E14.68 | 1 IP, 1 ED or 1 claim |
| Bacteremia | ICD-9-CM: 790.7  ICD-10-CA: R78.81 | 1 IP, 1 ED or 1 claim |
| Any fracture | See ICD codes for individual fractures listed below. | 1 IP, 1 ED or 1 claim for pelvis, foot, humerus, radius/ulna, upper limb, ankle, femur, hip, tibia fracture |
| Upper limb | ICD-9-CM: 810-819, 733.11, 733.12  ICD-10-CA: S42, S52, S62, T10, M84.39, M84.41-44, T02.20, T02.4, T07 | 1 IP, 1 ED or 1 claim |
| Foot | ICD-9-CM: 733.16, 825, 825.30, 825.20, 826, 826.0, 826.1, 824  ICD-10-CA: S92, S92.90, S92.20, S92.40, M84.37 | 1 IP, 1 ED or 1 claim |
| Hip | ICD-9-CM: 808, 820, 821, 733.98  ICD-10-CA: S32, S72.0, M84.35, M84.45, R57.9, R74.8, R81, R88.0, O28.9 | 1 IP, 1 ED or 1 claim |
| Radius/Ulna | ICD-9-CM: 813, 733.21  ICD-10-CA: S52.1, S52.3, S52.4, S52.6, M84.43, M85.4, M84.39, S52, M84.45, R01.2, R73.0, R82.3, R86.9, R93.8, R03.0 | 1 IP, 1 ED or 1 claim |
| Hand | ICD-9-CM: 814-817  ICD-10-CA: S62, S62.1-S62.4 | 1 IP, 1 ED or 1 claim |
| Ankle | ICD-9-CM: 824  ICD-10-CA: M84.47, T02.3, T02.5, S82 | 1 IP, 1 ED or 1 claim |
| Pelvis | ICD-9-CM: 733.95, 805, 806, 808  ICD-10-CM: S32, M84.35 | 1 IP, 1 ED or 1 claim |
| Tibia | ICD-9-CM: 823  ICD-10-CA: M84.46, M84.36, T02.3, T02.5, S82 | 1 IP, 1 ED or 1 claim |
| Humerus | ICD-9-CM: 812  ICD-10-CA: S42.2, S42.3, S42.4, S42.7, S42.8, M84.42, S42, R011, R700, R820, R195, R91, R292 | 1 IP, 1 ED or 1 claim |
| Femur | ICD-9-CM: 820, 821  ICD-10-CA: S720, M84.35, M84.45, S72.9 | 1 IP, 1 ED or 1 claim |
| All-cause mortality | - | 1 vital statistic record for death (any cause) |

Abbreviations: CCI: Canadian Classification of Health Intervention; CCP: Canadian Classification of Diagnostic, Therapeutic, and Surgical Procedures; CRRT: continuous renal replacement therapy; ED: emergency department; ESRD: end stage renal disease; HHF: hospitalization for heart failure; ICD 9-CM: The International Classification of Diseases, Ninth Revision, Clinical Modification; ICD-10-CA: International Statistical Classification of Diseases and Related Health Problems, 10th Revision; IP: inpatient.
